# Diagnostic biomarkers of ischemic stroke: strengths and limitations across blood, urine, and saliva

**DOI:** 10.3389/fneur.2026.1803045

**Published:** 2026-06-17

**Authors:** Begüm Utz, Pihla Miettinen, Ivette Bañuelos-Cabrera, Leonardo Lara-Valderrábano, Lasse Välimaa, Adrian Harel

**Affiliations:** Medicortex Finland Oyj, Turku, Finland

**Keywords:** biomarker, blood, diagnostic, ischemic stroke, review, saliva, stroke, urine

## Abstract

Ischemic stroke is a major cause of death and long-term disability worldwide. It requires quick medical intervention within a short timeframe to save brain tissue. While neuroimaging is the preferred method for diagnosis, it is often unavailable in prehospital and low-resource settings. This gap has led to a strong interest in finding biological markers that can quickly confirm ischemia and help differentiate it from hemorrhagic strokes and stroke mimics. This review summarizes the frequently studied diagnostic biomarkers found in blood, urine, and saliva, organizing them by molecular pathways such as neuronal injury, inflammation, hemostasis, and oxidative stress. Blood-based markers are currently the most advanced, with brain-specific proteins such as glial fibrillary acidic protein providing high specificity for early stroke subtyping. However, many other markers for neuronal injury have slow kinetics or poor specificity to differentiate stroke subtypes, which limits their immediate utility. Emerging research shows that brain-specific proteins and proteomic/metabolic signatures can be detected in urine and saliva, but these methods are still experimental and lack validation in larger acute patient groups. Overall, the current evidence indicates that no single biomarker or biofluid is sufficient for early ischemic stroke diagnosis. Future progress will likely depend on multi-marker strategies, standardized methods, and early-timepoint sampling.

## 1 Introduction

Stroke is one of the leading causes of death and disability worldwide. In 2021, stroke was estimated to cause nearly 7.3 million (95% UI 6.6–7.8) deaths and 160.5 million (147.8–171.6) disability-adjusted life-years globally (1). There is a large socioeconomic burden associated with stroke, due to costs related to long-term healthcare, rehabilitation, and loss of employment. This burden was estimated to be £29.67 billion in the United Kingdom and $214.61 billion in the United States in 2019 (2).

There are three main subtypes of stroke: ischemic stroke (IS), hemorrhagic stroke (HS), and subarachnoid hemorrhage (3). Among the stroke subtypes, IS is significantly more common than the other types, constituting an estimated 65.3% of the cases. In comparison, HS constitutes 28.8% and subarachnoid hemorrhage constitutes 5.8% of the stroke cases (1). IS arises from occlusion of a cerebral artery and reduction in cerebral perfusion, which then results in cerebral hypoxia, brain tissue damage, and necrosis. The term ‘ischemic penumbra’ refers to the area of the brain that remains viable for a limited time under ischemic conditions. If timely reperfusion is achieved, the viable tissue within the ischemic brain region can be saved, thus improving patient outcomes (4). To emphasize the importance of this critical time window, neurologists came up with the phrase ‘time is brain’ (5). The first 4.5 h following the IS onset constitute the treatment window for the administration of intravenous thrombolytics (tissue plasminogen activator—t-PA) (6). Given this narrow time window, it is extremely important to identify patients with stroke promptly and differentiate IS from other stroke subtypes and stroke mimics such as seizure, syncope, sepsis and migraine.

Currently, the diagnosis of stroke relies primarily on clinical assessment combined with neuroimaging methods such as non-contrast computed tomography (CT), magnetic resonance imaging (MRI), and vascular and perfusion imaging (7). The findings are used to confirm ischemia, exclude hemorrhage, and identify large-vessel occlusion. However, in the early stages of an IS, CT results might not indicate an abnormality. MRI performs better than CT in detecting acute ischemia and has been recommended as the primary choice for accurate diagnosis of patients suspected of an acute stroke (8). Unfortunately, neuroimaging is not always immediately available and can be time-consuming. The lack of the infrastructure and resources for advanced imaging is particularly severe in resource-limited settings. Consequently, there is a strong interest in identifying biomarkers that could support rapid diagnosis of IS and complement neuroimaging and clinical stroke assessment scales.

Biomarkers are biological molecules that are measurable indicators of a normal or a pathogenic process, or responses to an exposure or intervention (9). Specifically, a diagnostic biomarker detects or confirms the presence of a disease or a condition, or identifies an individual with a subtype of the disease (10). For IS diagnosis, it is critical that a suitable biomarker detects stroke soon after its onset and helps to differentiate it from HS and stroke mimics. The most common diagnostic fluid used for studying stroke biomarkers has been blood/plasma, however, urine and saliva are being studied as well. In the recent decades, numerous efforts have been put forth to identify early diagnostic biomarkers of IS. Recent reviews emphasize that, despite a large number of candidate biomarkers, none is yet endorsed in clinical guidelines for routine stroke diagnosis, which highlights both the promise and current limitations of this field.

**Figure 1:**
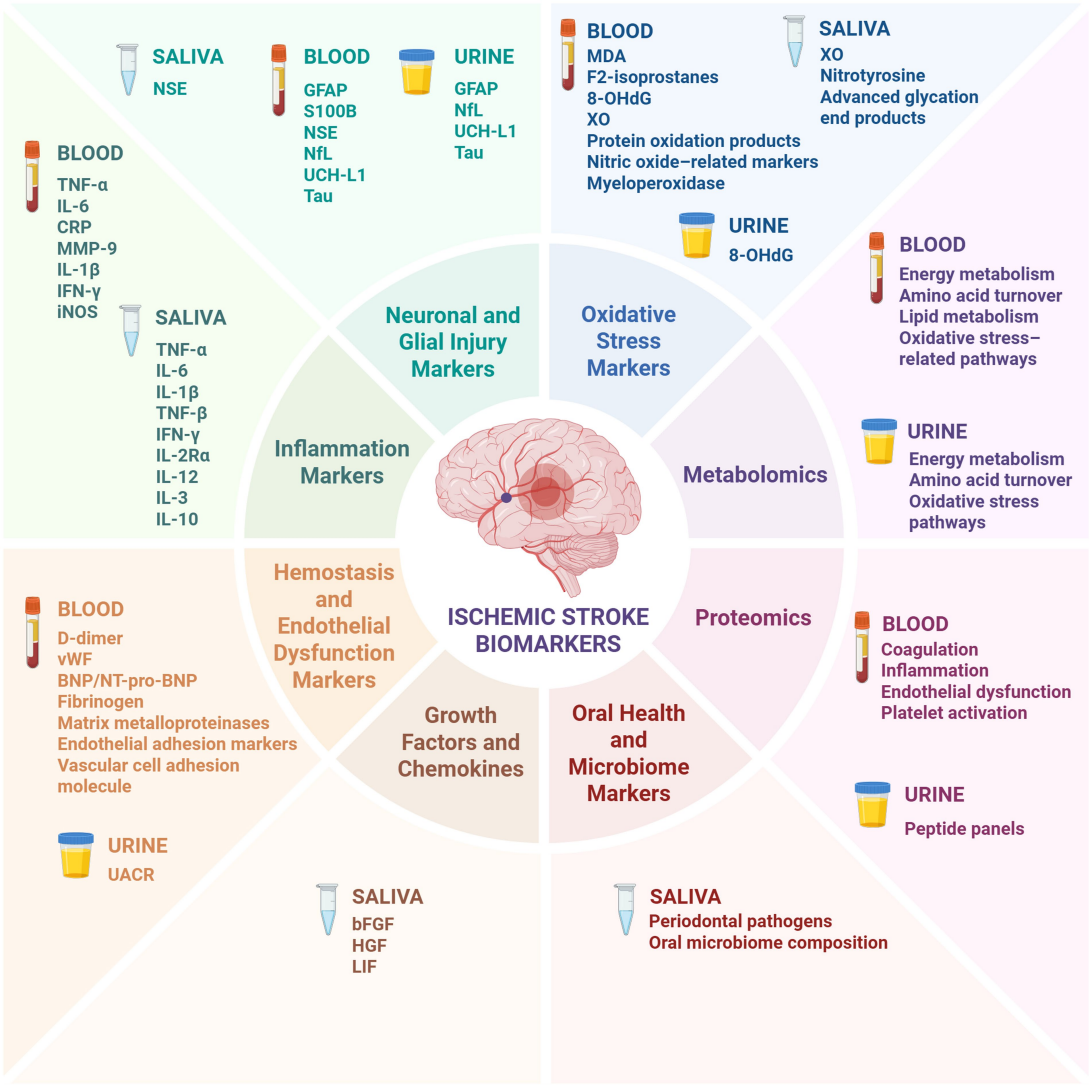
Major classes of ischemic stroke biomarkers studied in blood, urine, and saliva. The figure summarizes key biological pathways and the corresponding biomarkers identified in blood, urine and saliva. Blood-based biomarkers are the most extensively studied, while urinary and salivary biomarkers represent emerging non-invasive alternatives with more limited evidence. Created in BioRender. https://BioRender.com/gd7zev8.

In this review, we summarize evidence on diagnostic biomarkers most frequently investigated in the literature for IS, with a focus on key molecular pathways and different body fluids used (Figure 1). Blood-based biomarkers were selected using a descriptive PubMed search to assess how frequently individual biomarkers have been investigated in the context of IS biomarker research. Biomarkers included in this review have been repeatedly evaluated across independent studies and are supported by substantial bodies of evidence in human cohorts. Other candidate biomarkers, although biologically relevant, were not emphasized if they were less consistently studied. In contrast, because the literature on urinary and salivary biomarkers is considerably more limited, we aimed to include all available human studies examining diagnostic or biologically relevant markers in these body fluids. We further discuss the remaining gaps in evidence and outline priorities for future research.

## 2 Blood and plasma biomarkers for ischemic stroke diagnosis

Over the past two decades, the overwhelming majority of research on biomarkers of IS has focused on blood and plasma, resulting in hundreds of candidate molecules evaluated for their diagnostic potential (11–13). Most blood biomarkers under investigation originate from one or more pathobiological processes related to brain ischemia. These can be categorized as neuronal and glial injury, hemostasis and endothelial dysfunction, inflammation, oxidative stress, and omic-level signatures, including circulating microRNAs and metabolomic profiles. Many candidate markers have been studied as potential early diagnostic biomarkers of stroke and were assessed for their ability to differentiate between stroke subtypes. However, many of them either failed because their levels did not rise fast enough to help in acute stroke diagnosis and stroke subtyping, or they lacked the specificity to differentiate stroke from other conditions or between stroke subtypes (14).

Below, we examine the most promising diagnostic biomarkers of IS in blood.

### 2.1 Neuronal and glial injury markers

Neuronal and glial injury markers have been among the most extensively studied blood biomarkers in IS because they directly reflect structural damage to the central nervous system. During cerebral ischemia, interruption of blood flow leads to rapid energy failure, ionic imbalance, and excitotoxicity, ultimately causing neuronal depolarization, cytoskeletal breakdown, and glial activation (15). As the ischemic cascade progresses, both neurons and astrocytes undergo membrane disruption and cell death via necrosis in the ischemic core and apoptosis in the penumbra (16). This cell death results in the release of intracellular proteins into the extracellular space and eventually into the bloodstream. In parallel, ischemia-induced degradation of the neurovascular unit compromises blood–brain barrier (BBB) integrity, increasing its permeability and facilitating the passage of brain-derived proteins such as glial fibrillary acidic protein (GFAP), ubiquitin C-terminal hydrolase-L1 (UCH-L1), S100B, and neurofilament light chain (NfL) into circulation (17, 18).

*Glial fibrillary acidic protein (GFAP)* is an intermediate filament protein highly expressed in astrocytes and found primarily in the brain. As a biomarker of brain tissue damage in neurological conditions, serum GFAP has been studied in a number of clinical studies for the diagnosis of traumatic brain injury (TBI) and stroke (19–21). Studies found that serum GFAP levels rise within 6 h after symptom onset in patients with intracerebral hemorrhage (ICH), differentiating patients with ICH from those with IS and stroke mimics with a > 90% specificity (19, 22, 23). In IS, the serum GFAP levels rise more gradually over time, peaking between 2 to 5 days (24). GFAP shows strong potential as an early diagnostic biomarker for identifying ICH in prehospital settings. However, robust large-scale international studies are needed to develop and validate a GFAP assay capable of supporting rapid stroke diagnosis.

*S100B* is a calcium-binding protein predominantly expressed in astrocytes but also present in other glial and endothelial cells (25). S100B has long been considered to be a marker of brain damage, thus it has been studied extensively as an IS biomarker. Data from six longitudinal studies show that serum levels of S100B do not rise immediately following an IS but only peak at day 3 after stroke onset, thus eliminating its potential use as an early biomarker (26). Furthermore, S100B was found to have a low specificity for IS. However, elevated S100B levels have been consistently associated with infarct volume, hemorrhagic transformation risk, and poor neurological outcomes (26). In multi-marker panels, S100B contributes to prognostic modeling rather than early diagnostic accuracy. Thus, S100B is generally not considered a primary diagnostic biomarker.

*Neuron-specific enolase (NSE)* is a neuronal glycolytic enzyme that has been widely studied as a biomarker of neuronal injury (27). Following cerebral ischemia, NSE is released into the circulation with increases reported as early as within the first 4–6 h after symptom onset (28, 29). Several studies have shown that higher serum NSE concentrations in the acute phase are associated with larger infarction volumes, greater neurological severity, and worse short-term functional outcomes, supporting its role as a prognostic biomarker (30, 31). NSE levels were reported to peak around 48 h post-stroke (29, 31). In some patients, a delayed secondary peak in NSE has been linked to hemorrhagic transformation (32). Even though its levels rise early, NSE has limited utility for early stroke diagnosis, as serum levels do not reliably distinguish IS from stroke mimics and exhibit low specificity but high sensitivity similar to S100B (33).

*Neurofilament light chain (NfL)* is a cytoskeletal protein located in large-caliber myelinated axons, and it is released into the cerebrospinal fluid (CSF) and blood following axonal injury (34). Compared with other neuronal injury markers, NfL demonstrates slower kinetic dynamics. NfL levels increase gradually over days and often peak around 1–4 weeks post-stroke, reflecting ongoing axonal degeneration (35, 36). While the slow post-stroke rise of NfL limits its diagnostic utility, it remains a strong indicator of baseline stroke severity, infarct volume, and 90-day functional outcomes (35, 37, 38).

*Ubiquitin C-terminal hydrolase-L1 (UCH-L1)* is a neuron-specific deubiquitinase enzyme that is highly abundant in the brain (39). Enriched in neuronal cell bodies and axons, UCH-L1 was found to be required for the maintenance of axonal integrity (40). Numerous clinical studies found that UCH-L1 is released into the CSF and blood following acute neuronal injury in patients following stroke and TBI (21, 41, 42). UCH-L1 levels were found to be elevated as early as 2.5 h of stroke onset, and although UCH-L1 showed good diagnostic performance for identifying stroke, it was inferior in its diagnostic discrimination between IS and HS compared to GFAP (43). UCH-L1 may provide complementary value in biomarker panels, helping in capturing neuronal injury, but its individual performance remains suboptimal for stroke subtype differentiation. Furthermore, because UCH-L1 can also increase in other forms of acute brain injury, such as TBI, its stroke specificity may be limited in certain emergency cases.

*Tau* is an axonal microtubule-associated protein released following axonal injury in IS. In clinical studies, tau becomes detectable within hours after stroke onset, with increases reported as early as 6 h and peak concentrations occurring in 3–5 days later (44, 45). Elevated CSF and serum tau levels have been associated with larger infarct volumes, early neurological deterioration, and poorer functional outcomes, supporting its value as a marker of injury severity and prognosis (44–46). However, its limited specificity, due in part to overlap with other neurological and neurodegenerative conditions, constrain the utility of tau as a stand-alone early diagnostic biomarker for IS.

### 2.2 Hemostasis and endothelial dysfunction markers

IS is fundamentally a vascular event, arising from thrombus formation and embolic occlusion. As a result, biomarkers reflecting abnormalities in coagulation, platelet activation, fibrinolysis, vascular integrity, and endothelial dysfunction have been extensively investigated for their diagnostic potential in stroke (47). Cerebral ischemia triggers a cascade of hemostatic responses including activation of tissue factor pathways, thrombin generation, and fibrin turnover; while hypoxia and inflammatory mediators promote endothelial activation and increased vascular permeability. These processes lead to measurable changes in circulating molecules such as D-dimer, fibrinogen, von Willebrand factor, matrix metalloproteinases, and endothelial adhesion markers (48). While these markers are biologically relevant for stroke diagnosis, many of them have also been associated with systemic illnesses, which limits their specificity.

*D-dimer* is a fibrin degradation product that has been studied both as a diagnostic biomarker of stroke and as a means of subtyping IS (49). D-dimer levels are elevated early in IS compared with the healthy control population (50, 51). A meta-analysis by Ahmad et al., (52) identified that elevation of D-dimer levels within 24 h of stroke onset distinguished stroke from stroke mimics and controls. Furthermore, an early rise in the D-dimer levels was associated with large-vessel occlusions, which can be applied in prehospital triage to determine whether transport to a thrombectomy-capable center is required (53). Yang et al. (54) have demonstrated that D-dimer discriminates cerebral embolism from other IS subtypes. Despite this potential, D-dimer cannot reliably distinguish IS from HS or from stroke mimics, as D-dimer levels can be influenced by other diseases and conditions such as cancer, infection, atrial fibrillation, trauma, and advanced age. Thus, while D-dimer is not suited as a stand-alone diagnostic tool, it remains a valuable component of multi-marker approaches for early etiologic classification and triage in acute stroke care.

*B-type natriuretic peptide (BNP)* is synthesized as a high molecular weight precursor pro-BNP which is cleaved to release BNP and the amino-terminal fragment, *N-terminal pro-BNP (NT-pro-BNP)*. Both peptides are released from the cardiac ventricles in response to increased wall stress due to stretching and pressure (55). Elevated levels of BNP and NT-pro-BNP were reported in stroke, especially in cardioembolic stroke subtype (56). However, BNP and NT-pro-BNP do not reliably distinguish IS from HS or stroke mimics (57). These biomarkers can still be used in biomarker panels to identify a probable cardioembolic source and guide further evaluation, including prolonged cardiac rhythm monitoring and echocardiography.

*von Willebrand factor (vWF)* is a multimeric glycoprotein synthesized primarily by endothelial cells and megakaryocytes and plays a central role in platelet adhesion, aggregation, and thrombus stabilization. Comparing samples from suspected IS patients to healthy subjects, Lynch et al. (58) found that vWF levels, along with S100B, matrix metalloproteinase-9, and vascular cell adhesion molecule, were highly correlated with stroke. Similarly, higher vWF levels were found in IS patients than in control subjects in a more recent study (59). However, because vWF is also increased in systemic inflammation, cardiovascular disease, and acute stress states, its diagnostic specificity for stroke is limited.

### 2.3 Inflammation markers

Inflammation is a central component of IS pathophysiology. Within minutes following an ischemic brain damage, an inflammatory response is initiated through endothelial signaling, microglial activation, and peripheral immune cell recruitment (60). This, in turn, induces rapid upregulation of pro-inflammatory cytokines, chemokines, and acute-phase reactants, which contribute to BBB disruption, secondary neuronal injury, and infarct expansion. The circulating inflammation markers have thus been investigated for their potential diagnostic value in IS. These include pro-inflammatory mediators, including interleukin (IL)-1β, interferon-gamma (IFN-*γ*), tumor necrosis factor alpha (TNF-*α*), IL-6, inducible nitric oxide synthase (iNOS), and proteases such as matrix metalloproteinase-9 (MMP-9) (61).

*Interleukin-6 (IL-6)* is one of the most consistently elevated inflammatory cytokines in IS. Its levels rise rapidly within hours of symptom onset as part of the early immune response to cerebral ischemia (62). IL-6 is produced by activated microglia, astrocytes, endothelial cells, and infiltrating peripheral immune cells. Numerous clinical studies have shown that circulating IL-6 levels correlate with infarct volume, stroke severity, early neurological deterioration, and poor functional outcomes, highlighting its strong prognostic relevance (63). However, despite its early elevation, IL-6 has limited utility as a diagnostic biomarker because it lacks specificity for cerebral ischemia: IL-6 is also markedly increased in infection, trauma, malignancy, cardiovascular disease, and other acute inflammatory states commonly encountered in emergency settings (64).

Elevated IL-6 levels in circulation lead to the hepatic secretion of the *C-reactive protein (CRP)*. CRP is another inflammatory marker that is frequently elevated in IS, rising within 24 h; however, it lacks specificity for stroke, prohibiting its diagnostic use. Despite this, it remains associated with stroke risk and poor functional outcomes (65).

*Matrix metalloproteinase-9 (MMP-9)* is a zinc-dependent endopeptidase that plays a central role in extracellular matrix degradation and BBB disruption during IS. Following cerebral ischemia, MMP-9 is upregulated within 24 h of symptom onset. A recent meta-analysis of data from nearly 4,000 patients showed that MMP-9 has higher concentrations in IS patients compared to both stroke mimics and healthy controls, supporting its association with ischemic brain injury (66). However, considerable overlap with other inflammatory conditions, variability in sampling time, and lack of standardized cut-off values currently limit its specificity for IS diagnosis, underscoring the need for harmonized studies to better define its temporal kinetics and clinical utility.

### 2.4 Oxidative stress markers

Numerous studies have implicated oxidative stress in IS pathophysiology, arising from ischemia-induced mitochondrial dysfunction, molecular damage, excitotoxicity, and inflammation (67). Consequently, several oxidative stress markers have been investigated, most notably lipid peroxidation products such as malondialdehyde (MDA) and F2-isoprostanes, which are consistently elevated in IS, and correlate with infarct size and clinical severity (68). Markers of oxidative DNA damage, particularly 8-hydroxy-2′-deoxyguanosine (8-OHdG), have been detected in both plasma and urine, and are associated with poor functional outcomes and delayed recovery (69). Additional markers reported include protein oxidation products, nitric oxide–related markers, and enzymatic sources of reactive oxygen species including xanthine oxidase and myeloperoxidase (70). Despite strong mechanistic relevance, most oxidative stress markers lack sufficient specificity for IS diagnosis and are influenced by systemic inflammatory and cardiovascular conditions. Therefore, their principal value lies in prognostic assessment, pathophysiological insight, and inclusion in multi-marker or multi-omic approaches.

### 2.5 Blood proteomics and metabolomics

Blood-based proteomic profiling has provided an unbiased view of molecular changes in IS and identified several candidate diagnostic protein signatures. Human plasma and serum proteomics studies have consistently reported changes in proteins related to coagulation, inflammation, endothelial dysfunction, and platelet activation (71). Multi-protein panels derived from mass-spectrometry–based approaches have demonstrated the ability to distinguish IS from healthy controls, stroke mimics, transient ischemic attack, and HS. However, most studies relied on blood samples collected well beyond the therapeutic window, limiting their applicability for early diagnosis. In addition, many proteomic signatures require measurement of large numbers of proteins, posing practical challenges for clinical implementation. For a more detailed overview of proteomics studies on stroke biomarkers, readers are referred to the comprehensive review by Hochrainer and Yang (71).

Metabolomics is defined as the comprehensive analysis of metabolites in a biological sample. Metabolites have long been used to diagnose diseases related to metabolism, however, emerging studies have shown that metabolic changes in the brain can also lead to changes in metabolite levels in biological fluids, suggesting their potential use for diagnosis of neurological diseases (72). Blood-based metabolomic studies characterized systemic metabolic alterations associated with IS. Human metabolomics data have consistently demonstrated changes in energy metabolism, amino acid turnover, lipid metabolism, and oxidative stress–related pathways (73, 74). Several metabolite panels have shown potential to discriminate IS from controls and, in some studies, to differentiate stroke subtypes or predict functional outcome (73, 74). However, as with proteomic approaches, most metabolomic studies have been conducted outside the acute diagnostic window and reported varying metabolic signatures. Consequently, further validation is required before clinical translation of blood metabolomics markers.

## 3 Urinary biomarkers for ischemic stroke diagnosis

Urine represents a highly attractive body fluid for IS diagnosis. Its non-invasive collection, suitability for repeated sampling, and compatibility with prehospital or low-resource settings provide clear practical advantages over blood. Several studies have explored changes in metabolic, oxidative, endothelial, and neuroaxonal signals detectable in urine following cerebral ischemia. To date, most urinary IS biomarkers have been associated with varying clinical outcomes and patient prognosis, however, their diagnostic potential remains limited due to their lack of specificity and low abundance (75). In the recent years, new ultra-sensitive assay formats which enable femtogram-level detection of biomarkers have been developed which overcome the issue of low abundance of neurological biomarkers in different biofluids. These ultrasensitive assays achieve a sensitivity nearly 1,000 times more than the traditional ELISA, overcoming the analytical sensitivity barrier in using non-invasive body fluids for the detection of low abundance biomarkers. Below, we summarize the most prominent urinary markers of IS and different approaches used to identify them.

### 3.1 Neuronal and glial injury markers in urine

Recent studies have examined whether brain-specific proteins that are typically quantified in blood or CSF can also be detected in urine, enabling non-invasive biomarker detection after acute cerebral injury. Using ultrasensitive single-molecule arrays (Simoa®), one study showed that GFAP, NfL, UCH-L1, and total tau (t-tau) are quantitatively measurable in paired urine/serum samples from patients with IS and intracerebral hemorrhage within 96 h after symptom onset. Notably, urine concentrations were significantly higher in the acute stroke group compared to healthy controls. After correction for urine dilution (biomarker/creatinine ratios), NfL and t-tau showed the strongest effects (76). A more recent study also used ultrasensitive single-molecule arrays to detect brain injury biomarkers in urine from patients with IS and intracerebral hemorrhage, and found that higher urine GFAP (and to a lesser extent urine NfL and t-tau) levels in patients correlated with injury burden and in-hospital death or death by 3 months (77). However, urinary biomarker levels failed to distinguish between the two stroke subtypes, which was attributed to the relatively late sampling window, with urine collected up to 96 h after symptom onset. Another recent study focused specifically on urinary UCH-L1 across multiple acute brain injury (ABI) types (including IS) and quantified UCH-L1 using ELISA. This study found markedly elevated urinary UCH-L1 in ABI patients compared with healthy controls, with excellent discrimination at 36 h post-injury sampling timepoint (AUC ~ 0.98) (78).

Taken together, these studies support the technical feasibility of measuring brain injury markers in urine and suggest that they may provide supportive diagnostic information, particularly in distinguishing acute brain injury from healthy states. However, further research with larger sample sizes and earlier timepoints is needed to determine the diagnostic value of these biomarkers for stroke. As a result, current evidence supports the use of urinary brain injury markers primarily for assessing injury burden and prognosis rather than for early diagnosis of IS.

### 3.2 Urinary proteomics and metabolomics

In terms of diagnostic evidence, proteomics analysis of urine samples from IS patients and controls has showed the highest specificity and overall diagnostic performance. Dawson et al. (79) collected urine samples from stroke patients within 24 h of symptom onset and developed a urinary proteomic biomarker panel based on 35 peptides which distinguished acute stroke from controls, particularly in patients presenting with mild or atypical symptoms. Although these findings were promising, the proposed 35-peptide panel has not yet been evaluated in larger cohorts. Moreover, despite substantial advances in mass spectrometry technologies over the past decade, urinary proteomic profiling in IS has not been revisited in more recent studies, representing a notable gap in stroke biomarker research.

Urinary metabolomics have been shown to capture metabolic responses to cerebral ischemia. Using proton nuclear magnetic resonance (^1H-NMR) spectroscopy, urinary metabolite profiles were able to distinguish patients with cerebral infarction from control subjects, indicating that IS is associated with a distinct metabolic pattern in urine (80). The altered metabolites reported in this study were mainly related to energy metabolism, amino acid turnover, and oxidative stress pathways. Subsequent studies expanded on these findings. Sidorov et al. (81) compared acute (72 h) and chronic (3–5.2 months) stroke stages and showed that two urine metabolites, glycine and acetylcarnitine, had significantly different concentrations in the acute stage. A more recent study used NMR-driven metabolomic profiling and identified a subset of metabolites (pseudouridine, 4-hydroxy-3-methoxymandelate, and inosine) with a high diagnostic accuracy in distinguishing between acute and chronic stroke samples (82). A 2025 systematic review of metabolomics in IS concluded that urine-based metabolite analyses are promising but emphasized substantial heterogeneity in analytical platforms, normalization strategies, and validation cohorts as major barriers to diagnostic implementation (73). Overall, urinary metabolomics holds diagnostic promise, but no consensus metabolite panel has yet been validated for acute IS diagnosis.

### 3.3 Oxidative DNA damage marker: 8-hydroxy-2′-deoxyguanosine (8-OHdG)

The most extensively studied single urinary biomarker in IS is 8-hydroxy-2′-deoxyguanosine (8-OHdG), which is a stable marker of oxidative DNA damage. A 2012 study reported elevated urinary 8-OHdG levels in IS, with associations to stroke subtype and functional outcomes (83). Subsequent work demonstrated that patients with higher 8-OHdG levels had lower upper-limb motor function and muscle power. Decreasing 8-OHdG levels following rehabilitation predicted better functional recovery (84). Despite its prognostic value, urinary 8-OHdG lacks sufficient specificity for diagnostic use, as its levels are influenced by systemic inflammation, infection, malignancy, and cardiovascular disease (85). Consequently, 8-OHdG is better suited as a marker of oxidative injury burden and prognosis rather than a diagnostic indicator for IS.

### 3.4 Urinary albumin-to-creatinine ratio (UACR)

Urinary albumin, also known as albuminuria, is a sign of kidney dysfunction and has been linked to cardiovascular events. Several studies have found elevated UACR in IS, and this increase has been associated with elevated risk of incident IS and poor functional outcomes after stroke (86–88). However, UACR lacks the specificity for acute cerebral ischemia, limiting its diagnostic utility.

## 4 Salivary biomarkers for ischemic stroke diagnosis

Saliva has gained recognition as a valuable, non-invasive biofluid for biomarker discovery due to its ease of collection, repeatability, and close physiological linkage to systemic circulation (89). Salivary glands are highly vascularized, and many circulating molecules can enter saliva through passive diffusion, ultrafiltration, or via gingival crevicular fluid, particularly under conditions of BBB disruption and systemic inflammation following cerebral ischemia (90). Experimental and clinical studies demonstrate that inflammatory mediators, oxidative stress products, metabolites, and even brain-derived proteins measurable in blood can also be detected in saliva, supporting its feasibility as a diagnostic medium for IS (107, 75). However, salivary biomarker research in stroke remains relatively early-stage, with limited number of studies. Furthermore, most studies using saliva have been conducted outside the acute diagnostic window. Thus, currently, saliva for stroke diagnosis can be best regarded as an experimental body fluid.

### 4.1 Inflammatory cytokines, chemokines, and growth factors

Inflammation is a prominent feature of IS pathophysiology, and several studies have demonstrated altered salivary inflammatory profiles in IS patients. The first study to investigate salivary cytokines in IS reported that key pro-inflammatory cytokines such as TNF-*α* and IL-6 were significantly elevated in both non-stimulated and stimulated saliva of IS patients compared with matched controls in the subacute phase between 30 and 35 days after the incident (91). In addition, anti-inflammatory cytokine IL-10 was found to be lower in the stroke samples compared to the controls. Building on this, the same group conducted a comprehensive screening assessment of different groups of cytokines (pro-inflammatory, anti-inflammatory, and cytokines released from T helper cells, Th1, Th2, and Th17) in unstimulated saliva from IS patients and matched healthy individuals. This work demonstrated significantly higher levels of multiple classes of cytokines (e.g., proinflammatory cytokines IL-1*β*, TNF-α, TNF-β, IFN-*γ*; anti-inflammatory cytokine IL-2Rα; Th1 cytokine IL-12, and Th2 cytokine IL-6) in stroke patients (92).

Extending beyond classical cytokines, the same group also examined a broader panel of salivary chemokines and growth factors, identifying significant disruptions in factors such as basic fibroblast growth factor (bFGF), hepatocyte growth factor (HGF), interleukin-3 (IL-3), and leukemia inhibitory factor (LIF) in IS patients relative to controls (93). Receiver operating characteristic analysis indicated that several of the growth factors (bFGF, HGF, IL-3, LIF) could discriminate between patients and controls with high sensitivity and specificity, suggesting that salivary inflammatory and growth factor signatures may provide diagnostic information. However, these studies were done with samples from patients in the subacute phase (10–35 days following the stroke incident), and further investigations into earlier levels of these markers are needed.

### 4.2 Salivary gland dysfunction and oxidative stress markers

Stroke has been associated with salivary gland dysfunction and redox imbalance, providing a mechanistic basis for salivary oxidative stress markers. In a case–control study, stroke patients with reduced salivary flow exhibited increased salivary protein glycoxidation and nitrosative stress, including elevated advanced glycation end products and nitrotyrosine, with changes most pronounced in non-stimulated saliva (94).

Building on this, the same group examined salivary redox and inflammatory biomarkers in stroke patients and found salivary xanthine oxidase (XO), which is a key enzymatic source of reactive oxygen species, to be significantly elevated in non-stimulated saliva of IS patients compared with HS and controls (95). Although ROC analyses for XO indicated good discriminatory performance under controlled oral-health conditions, as sampling was performed in the subacute phase (between 30 and 35 days since the stroke incident), the diagnostic relevance of salivary XO for early IS identification remains to be validated.

### 4.3 Oral health-related markers and periodontal inflammation

Oral health impairment and periodontal inflammation are common in IS. Case–control studies have shown higher salivary and systemic markers of periodontitis and increased detection of periodontal pathogens in stroke patients compared with controls, consistent with advanced periodontal disease and elevated systemic inflammatory burden (96). Reduced salivary flow, poorer oral hygiene, and increased salivary oxidative stress have also been reported in stroke cohorts, reflecting salivary gland dysfunction and post-stroke functional impairment (97, 98).

More recently, alterations in oral microbiome composition and diversity have been observed in both IS and transient ischemic attack, with microbial signatures associated with stroke severity and functional outcomes (99, 100). Collectively, these findings indicate that oral health–related markers primarily reflect pre-existing oral disease and systemic inflammation rather than acute cerebral ischemia, and should therefore be considered important confounders, not diagnostic biomarkers, in salivary stroke research.

### 4.4 Neuronal and glial injury markers in saliva

Neuronal and glial injury markers are among the most promising candidates for stroke diagnosis, as they directly reflect structural damage to brain tissue. However, investigations of these biomarkers in saliva remain limited. A 2009 study investigated the serum and saliva levels of NSE in patients with IS, high-risk group patients and healthy controls, and found that salivary NSE concentrations were significantly higher in patients compared to healthy controls (101). In contrast, other neuronal and glial injury markers, such as S100B, have not yet been systematically evaluated in salivary samples from IS patients. Although elevated salivary S100B levels have been reported in other conditions associated with brain tissue damage, such as TBI, their diagnostic potential for IS remains unexplored (102, 103).

## 5 Discussion

Rapid and accurate diagnosis of IS is critical, as timely initiation of reperfusion therapies remains the most effective strategy to reduce brain injury and improve functional outcomes. For early diagnosis, clinical examination alone may be insufficient to differentiate IS from HS and stroke mimics. To support the diagnosis, neuroimaging methods are used, however, access to advanced imaging may not be immediately available in prehospital settings or resource-limited environments. Thus, there is a strong unmet need for early diagnostic biomarkers that can support rapid stroke diagnosis, differentiating IS from HS and stroke mimics. As outlined in this review, research in the last few decades has discovered many candidate biomarkers for IS, however, their clinical use as early diagnostic tools remains limited (Table 1).

**Table 1:** Overview of ischemic stroke biomarkers by pathophysiological class, biofluid, and key limitations.

Biomarker	Type	Pathophysiological class	Blood/Plasma	Urine	Saliva	Key limitations
Neuronal and glial injury markers						
GFAP	Diagnostic	Glial injury	Yes Rises within 6 h in HS (19)	Yes Tested at 96 h (76)	NR	Slow kinetics in IS; peaking in blood in 2–5 days (24, 45)
S100B	Prognostic	Glial injury	Yes Peaks at day 3 (26)	NR	NR	Poor specificity
NSE	Diagnostic/Prognostic	Neuronal injury	Yes Rises within 4–6 h (28, 29)	NR	Yes (101)	Poor specificity
NfL	Prognostic	Axonal injury	Yes Peaks at weeks 1–4 (35, 36)	Yes Tested at 96 h (76)	NR	Slow kinetics
UCH-L1	Diagnostic	Neuronal injury	Yes Rises within 2.5 h (43)	Yes Tested at 36 h (78)	NR	Poor specificity
Tau	Diagnostic/Prognostic	Neuronal injury	Yes Rises within 6 h (44, 76)	Yes Tested at 96 h (76)	NR	Poor specificity
Hemostasis and endothelial dysfunction markers						
D-dimer	Diagnostic	Hemostasis	Yes Rises within 24 h (52)	NR	NR	Poor specificity
BNP / NT-pro-BNP	Diagnostic/Prognostic	Cardioembolic signaling	Yes (56)	NR	NR	Poor specificity
vWF	Diagnostic	Endothelial dysfunction	Yes (59)	NR	NR	Poor specificity
UACR	Prognostic	Endothelial dysfunction	NR	Yes (86–88)	NR	Poor specificity
Inflammation markers						
IL-6	Prognostic	Inflammation	Yes Rises within hrs (62)	NR	Yes Tested at days 30–35 (91)	Poor specificity
CRP	Predictive/Prognostic	Inflammation	Yes Rises within 24 h (65)	NR	NR	Poor specificity
MMP-9	Diagnostic	Inflammation/BBB disruption	Yes Rises within 24 h (66)	NR	NR	Poor specificity
Growth factors (bFGF, HGF, IL-3, LIF)	Diagnostic	Repair / angiogenesis	NR	NR	Yes Tested at days 10–13 (93)	Lacks acute phase data
Oxidative stress markers						
MDA/F2-isoprostanes	Diagnostic/Prognostic	Lipid peroxidation	Yes (68)	NR	NR	Poor specificity
8-OHdG	Prognostic	Oxidative DNA damage	Yes (69)	Yes (25, 69)	NR	Poor specificity
XO	Diagnostic	ROS generation	Yes (70)	NR	Yes Tested at days 30–35 (95)	Lacks acute phase data
Proteomics						
Protein panels	Diagnostic	Multi-pathway	Yes Reviewed by (71)	Yes Tested at 24 h (79)	NR	Limited validation
Metabolomics						
Metabolite panels	Diagnostic/Prognostic	Multi-pathway	Yes (73, 74)	Yes Tested at 72 h and 3–5.2 months (81)	NR	Limited validation
Oral health-related markers						
Oral pathogens and related markers	Predictive/Prognostic	Oral-systemic inflammation	NR	NR	Yes (96–98)	Poor specificity
Oral microbial signatures	Predictive/Prognostic	Oral microbiome composition and diversity	NR	NR	Yes (99, 100)	Poor specificity

‘NR’ means ‘none reported’, meaning that no studies have been found in the literature. For an in-depth summary of diagnostic performance of most of these biomarkers (sensitivity, specificity, optimal time windows, and cut-off values), we refer the reader to the article by Dagonnier et al. (14). GFAP: glial fibrillar acidic protein; HS: hemorrhagic stroke; IS: ischemic stroke; NSE: neuron-specific enolase; NfL: neurofilament light chain; UCH-L1: ubiquitin C-terminal hydrolase-L1; BNP: B-type natriuretic peptide; NT-pro-BNP: N-terminal pro-BNP; vWF: von Willebrand factor; UACR: urinary albumin-to-creatine ratio; IL-6: interleukin-6; CRP: C-reactive protein; MMP-9: matrix metalloproteinase-9; BBB: blood brain barrier; bFGF: basic fibroblast growth factor; HGF: hepatocyte growth factor; IL-3: interleukin-3; LIF: leukemia inhibitory factor; MDA: malondialdehyde; 8-OHdG: 8-hydroxy-2′-deoxyguanosine; XO: xanthine oxidase; ROS: reactive oxygen species.

Across the three biofluids examined, blood-based biomarkers are the most advanced with respect to their diagnostic relevance. Brain-derived biomarkers such as GFAP, UCH-L1, S100B, and NfL reflect neuronal and glial injury and show consistent associations with stroke presence, severity, and outcome (14). However, their temporal kinetics differ substantially, and most rise too late to serve as stand-alone early diagnostic tools for IS. GFAP has emerged as a notable exception for early discrimination of intracerebral hemorrhage from IS, as its levels rise within 6 h in patients with ICH (19). Vascular, hemostatic, and inflammatory markers including D-dimer, BNP/NT-pro-BNP, vWF, IL-6, CRP, and MMP-9, often increase earlier but lack specificity for cerebral ischemia and are heavily confounded by systemic conditions (104). These observations reinforce the conclusion that single blood biomarkers do not provide high enough specificity for early IS diagnosis, however, combined multi-marker approaches are more likely to succeed.

Urine and saliva can be attractive non-invasive alternatives for diagnostics, particularly for prehospital settings. However, their diagnostic value for IS remains to be proven. In urine, brain-specific proteins such as GFAP, NfL, UCH-L1, and t-tau were detectable using ultrasensitive single-molecule arrays and their concentrations were higher in the acute stroke group than in healthy controls (76, 77). However, these samples were taken up to 96 h of stroke onset, thus, further research is needed to determine their concentrations in the acute phase. In addition, biomarker concentrations in these body fluids are substantially lower than in blood, necessitating the use of ultrasensitive analytical assays, which may limit feasibility for rapid point-of-care implementation. The strongest early diagnostic signal for IS in urine has emerged from proteomic analysis (79). However, these findings have not yet been validated in larger cohorts and are currently far from the capabilities of routine diagnostic tests. To advance urine-based diagnostics, future studies should focus on adapting and improving high-sensitivity technologies, such as single-molecule arrays, microfluidic platforms, mass spectrometry, and biosensors, for reliable detection of low-abundance brain-derived biomarkers in non-invasive fluids and eventual point-of-care use.

Salivary biomarkers further illustrate both the promise and the challenges of alternative body fluids. Changes in inflammatory cytokines, chemokines, growth factors, oxidative stress enzymes, and metabolomic and microbiome signatures have been reported, however, most studies with saliva used samples collected in the subacute phase of stroke (91, 93, 95). Therefore, their diagnostic ability in the acute phase remains to be explored. At the same time, reports show that oral health, periodontal disease, salivary gland dysfunction, and microbiome composition, may also influence stroke risk as well as salivary profiles of biomarkers. At present, saliva is best positioned as a complementary body fluid for stroke risk and prognosis, and more work is needed to determine its potential as a diagnostic tool for IS.

A common theme emerging from this review is the importance of biomarker kinetics. Biomarkers differ markedly in their onset, peak, and duration after stroke, and many studies have sampled outside the narrow time window in which diagnostic decisions about thrombolysis or thrombectomy must be made. Early-rising inflammatory or hemostatic markers may offer speed but lack specificity, whereas brain-specific injury markers offer specificity but rise later. This temporal mismatch has likely contributed to repeated failures in clinical translation. Systematic investigation of biomarker time courses, particularly within the first hours after symptom onset, is therefore essential. Furthermore, future studies which validate candidate biomarkers in large, multicenter, longitudinal stroke cohorts with long-term follow-up are needed to reliably define diagnostic sensitivity, specificity, and kinetic profiles across acute time windows. In addition, biomarker panels which combine brain-specific injury markers with other biomarker classes may provide better diagnostic value, improving specificity and stroke subtype discrimination. Advances in detection technologies, point-of-care platforms, and multiplex biosensors offer opportunities to realize such multi-panel approaches.

Another important future direction is to combine fluid biomarkers with clinical information and imaging data in integrated diagnostic models. Biomarkers may improve decision-making when used together with stroke severity scales, vascular risk factors, symptom timing, and CT or MRI findings. Recent studies show that artificial intelligence and machine learning can support stroke imaging analysis, including lesion detection, estimation of stroke onset time, treatment selection, and outcome prediction (105). Similar approaches could be used to combine biomarker results with clinical and imaging features to improve early diagnosis, stroke subtyping, triage, and prognosis evaluation. For example, combining blood biomarkers such as D-dimer and GFAP with clinical stroke scales has been reported to improve detection of large-vessel occlusion, suggesting that biomarkers may be most useful as part of practical triage algorithms rather than as stand-alone tests (106). Future studies should therefore focus on developing and validating these combined models in hyperacute and prehospital cohorts, where rapid and accurate decision-making is most needed.

In conclusion, while diagnostic biomarkers for IS across blood, urine, and saliva show substantial biological promise, none have yet achieved the performance, robustness, and validation required for routine clinical use.
